# Saquayamycin B_1_ Suppresses Proliferation, Invasion, and Migration by Inhibiting PI3K/AKT Signaling Pathway in Human Colorectal Cancer Cells

**DOI:** 10.3390/md20090570

**Published:** 2022-09-07

**Authors:** Jianjiang Li, Ningning Han, Hao Zhang, Xiaoyu Xie, Yaoyao Zhu, E Zhang, Jiahui Ma, Chuangeng Shang, Mengxiong Yin, Weidong Xie, Xia Li

**Affiliations:** 1 Marine College, Shandong University, Weihai 264209, China; 2 School of Pharmaceutical Sciences, Shandong University, Jinan 250012, China

**Keywords:** saquayamycin B_1_, colorectal cancer, PI3K/AKT, EMT, apoptosis, structure–activity relationship

## Abstract

Moromycin B (Mor B), saquayamycin B_1_ (Saq B_1_), saquayamycin B (Saq B), and landomycin N (Lan N), four angucyclines produced by the marine-derived actinomycete *Streptomyces* sp., are a class of polyketone compounds containing benzanthracene. Here, the structure–activity relationship of these four compounds was analyzed in human colorectal cancer (CRC) cells. Saq B_1_, which showed the strongest cytotoxicity with an IC_50_ of 0.18–0.84 µM for CRC cells in MTT assays, was employed to test underlying mechanisms of action in SW480 and SW620 cells (two invasive CRC cell lines). Our results showed that Saq B_1_ inhibited CRC cell proliferation in a dose- and time-dependent manner. Notably, lower cytotoxicity was measured in normal human hepatocyte cells (QSG-7701). Furthermore, we observed proapoptosis, antimigration, and anti-invasion activities of Saq B_1_ in CRC cells. At the same time, the protein and mRNA expression of important markers related to the epithelial–mesenchymal transition (EMT) and apoptosis changed, including N-cadherin, E-cadherin, and Bcl-2, in Saq B_1_-treated CRC cells. Surprisingly, the PI3K/AKT signaling pathway was shown to be involved in Saq B_1_-induced apoptosis, and in inhibiting invasion and migration. Computer docking models also suggested that Saq B_1_ might bind to PI3Kα. Collectively, these results indicate that Saq B_1_ effectively inhibited growth and decreased the motor ability of CRC cells by regulating the PI3K/AKT signaling pathway, which provides more possibilities for the development of drugs in the treatment of CRC.

## 1. Introduction

Colorectal cancer (CRC) is one of the most common malignancies of the digestive tract. Globally, CRC ranked third in terms of incidence (10.0%) and second in terms of mortality (9.4%) after lung cancer in 2020 [1]. In the United States, according to American Cancer Society research, approximately 106,180 people will be diagnosed with CRC in 2022, while more than 52,580 people will die from the disease [2]. CRC has the characteristics of high invasion and metastasis, which explain why it is difficult to cure. Some studies have reported that the 5-year relative survival rate of CRC is 90% in localized diseases, but this plummets to about 14% in patients with metastasis to distal organs [3]. In addition, approximately 25% of patients will already have distal metastasis at initial diagnosis, and 50% of CRC patients will eventually develop metastatic disease [4]. Currently, surgery remains the primary treatment for early CRC. However, due to the high heterogeneity, insidious onset, and rapid development of the disease, many CRC patients are diagnosed with invasion and metastasis in the middle and late stages of the disease, which often require combined radiotherapy and chemotherapy, and other treatment methods [5]. However, given the poor surgical recovery, adverse drug reactions, and drug resistance, novel drugs are urgently needed for the treatment of CRC.

The PI3K/AKT signaling pathway plays an important role in the growth, proliferation, angiogenesis, invasion, and migration of tumor cells, and has been identified as a key target for tumor-targeted therapy in recent years [6,7]. Abnormal activation of the PI3K/AKT signaling pathway confers proliferative advantages to tumor cells, and *PIK3CA* mutations are one of the most common types of CRC. The *PIK3CA* gene encodes the PI3K protein. Most known *PIK3CA* mutations (>80%) are in exon 9 or 20, encoding the helix domain and the kinase domain, respectively, and tumors with combined mutations are more severe than tumors containing only one of the mutations [8,9]. PI3K is activated to produce the second messenger phosphatidylinositol-3,4,5-trisphosphate (PIP3), which binds to the N-terminal PH domain of AKT to transfer AKT from the cytoplasm to the cell membrane, thereby promoting AKT phosphorylation. Phosphorylated-AKT (p-AKT) affects the expression of downstream target proteins such as Bad, NF-κB, GSK3, mTOR, and P53, thereby mediating various biological functions such as cell growth and proliferation [10]. P-AKT is considered a therapeutic target for malignant tumors [11]. At the same time, studies have reported a high positive expression rate of p-AKT in advanced CRC metastasis, suggesting that p-AKT protein plays an important role in the occurrence and development of CRC [12]. Therefore, inhibition of PI3K expression in tumor cells has become a key target to reduce the abnormal proliferation and migration of tumor cells.

Angucyclines are a class Ⅱ aromatic polyketone compound containing benzanthracene, which is mainly produced by the actinomycete *Streptomyces* sp. [13]. Its molecular framework is synthesized by type Ⅱ polyketide synthase using one promoter unit (acetyl-CoA) and nine extension units (malonyl-CoA) [14]. Compared with anthracyclines, which belong to the family of polycyclic aromatic polyketides, angucyclines showed higher antitumor activity due to their complex and variable structure, and these natural products have a wide range of potential applications in the medical field [15]. At present, studies have shown that angucyclines can induce tumor cell death in various ways, such as mitochondrial damage, inhibition of topoisomerase activity, and oxidative stress reactions, and thus play an antitumor role [16,17,18]. Saquayamycin B_1_ (Saq B_1_), one of the angucyclines, has a strong inhibitory effect on a variety of cell lines, such as PC3 and A549 cells, but its specific mechanism has not been studied [19,20]. Our previous study showed that saquayamycin B, the same type of compound as Saq B_1_, can induce apoptosis of liver cancer cells SMMC-7721 and inhibit the invasion and metastasis of the triple-negative breast cancer cell line MDA-MB-231, suggesting that Saq B_1_ may also have similar effects [15,21].

In this study, we aimed to analyze the structure–activity relationship of four angucyclines, further explore the anti-CRC effect of Saq B_1_, and evaluate the underlying molecular mechanism. Relevant experiments have proven that Saq B_1_ can not only induce apoptosis but also inhibit invasion and metastasis in CRC cells through the PI3K/AKT signaling pathway, which may provide a new avenue for the treatment of CRC.

## 2. Results

### 2.1. Saq B_1_ Inhibits the Proliferation of CRC Cells

**Figure 1:**
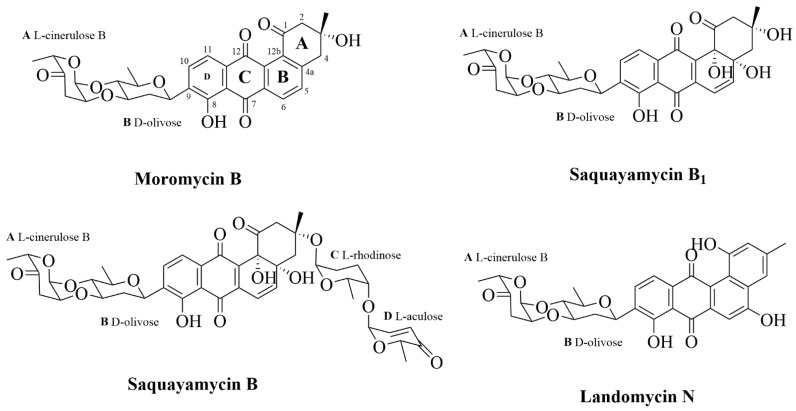
The chemical structure of Mor B, Saq B_1_, Saq B, and Lan N.

**Figure 2:**
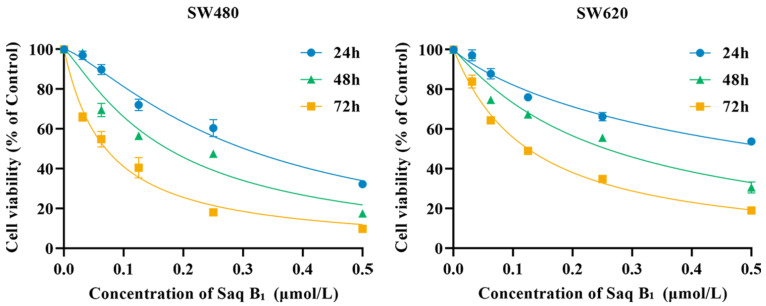
Saq B_1_ dose- and time-dependently inhibited the proliferation of SW480 and SW620 cells. Cells were treated with various concentrations of Saq B_1_ (0 to 0.5 μmol/L) from 24 h to 72 h.

To compare the effects of four angucyclines (moromycin B (Mor B), saquayamycin B_1_ (Saq B_1_), saquayamycin B (Saq B), and landomycin N (Lan N)) (Figure 1) and doxorubicin on CRC cells, we first performed MTT analysis in several subtypes of CRC cells (SW480, SW620, LoVo, and HT-29) and a noncancer cell line (QSG-7701). According to Table 1, Mor B, Saq B_1_, and Saq B showed greater inhibitory effects on CRC cells than doxorubicin, which is an anthracycline in the type Ⅱ polyketides. Surprisingly, among the four angucyclines, Saq B_1_ showed better activity against CRC cells. SW480 cells were more sensitive to Saq B_1_ than SW620 cells, and Saq B_1_ showed lower cytotoxicity in normal human liver cells. After 48 h of treatment, the final half-maximal inhibitory concentration (IC_50_) values of Saq B_1_ on SW480, SW620, LoVo, HT-29, and QSG-7701 cells were 0.18 ± 0.01 µmol/L, 0.26 ± 0.03 µmol/L, 0.63 ± 0.07 µmol/L, 0.84 ± 0.05 µmol/L, and 1.57 ± 0.12 µmol/L, respectively. Further experiments showed that Saq B_1_ inhibited the proliferation of SW480 and SW620 cells in a dose- and time-dependent manner (Figure 2). Therefore, SW480 was used to study the main mechanism of Saq B_1_, and SW620 cells were used for additional verification.

### 2.2. Saq B_1_ Regulates the PI3K/AKT Pathway

**Figure 3:**
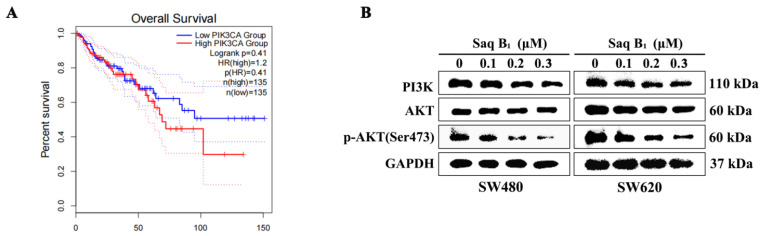

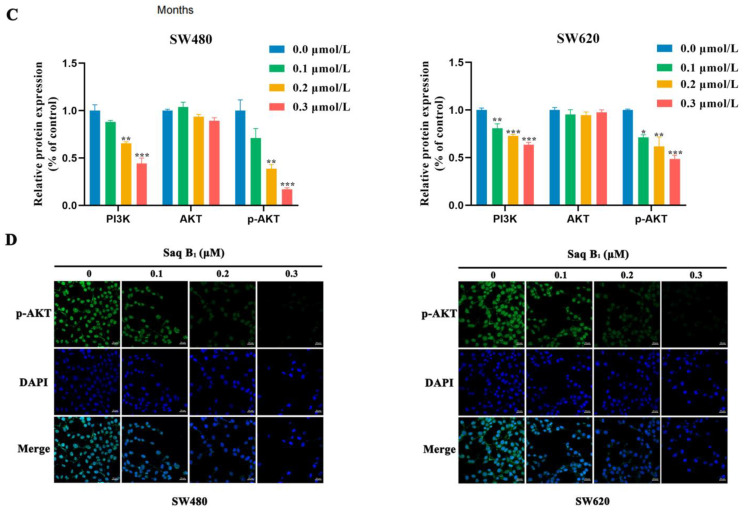
Effect of Saq B_1_ against the PI3K/AKT pathway in SW480 and SW620 cells. (**A**) The overall survival rate of patients with high or low *PIK3CA* expression was assessed via Kaplan–Meier survival analysis. (**B**,**C**) Western blot analysis was used to investigate the effects of Saq B_1_ on PI3K, AKT, and p-AKT proteins. (**D**) The expression of p-AKT was observed by immunofluorescence staining. Representative images of each condition are shown; p-AKT (green) and DAPI (blue). Scale bars = 20 µm. The level of intracellular p-AKT decreased, which was consistent with Western blot results. * *p* < 0.05, ** *p* < 0.01, *** *p* < 0.001.

Under normal conditions, PI3K/AKT activation is tightly controlled and depends on extracellular growth signals and the uptake of other substances such as amino acids and glucose [22]. There are high-frequency mutations in the PIK3CA gene in CRC, of which the activation mutation accounts for more than 80%. The activation mutation can make the PI3K protein remain activated independently of upstream signals. Through GEPIA database analysis, we found that patients with high expression of the PIK3CA gene had a lower survival rate than patients with lower expression (Figure 3A). The PI3K/AKT signaling pathway is extensively activated, which is closely related to tumorigenesis and development. As can be seen from Figure 3B,C, the protein expression levels of PI3K and p-AKT decreased after the treatment of SW480 and SW620 cells with 0.1 μM, 0.2 μM, and 0.3 μM of Saq B_1_, and the protein expression level of AKT was basically unchanged, which was consistent with our ideal result. Immunofluorescence staining showed that p-AKT, an important oncogenic effector in this pathway, was inhibited after Saq B_1_ treatment (Figure 3D).

**Figure 4:**
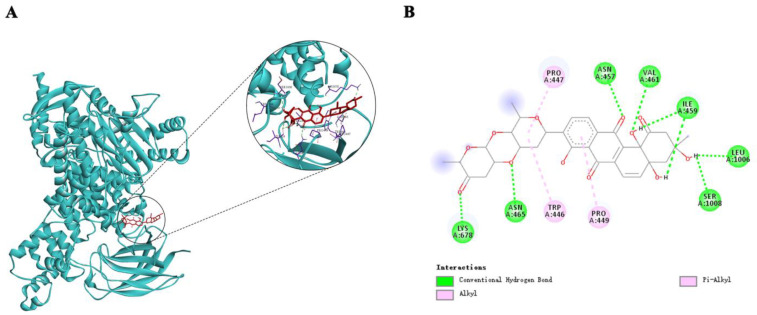
Theoretical binding mode of Saq B_1_ to PI3Kα protein. (**A**) AutoDockTools software was used to obtain the optimal docking model of Saq B_1_ to the PI3Kα protein, and the image obtained was a 3D panorama of the optimal binding mode. (The chemical structure of Saq B_1_ is represented by a red rod-shaped structure, the blue band represents the spatial structure of the PI3Kα protein, and the purple rod-shaped structure represents the amino acid residue bound to Saq B_1_). The lowest binding energy configuration is −8.34 kcal/mol. (**B**) The simplified two-dimensional interaction model is presented, in which some carbonyl and hydroxyl groups of Saq B_1_ are hydrogen-bonded to amino acid residues, while the D-olivose and benzene ring are bonded to amino acid residues Pro447, Trp446, and Pro449 in the form of alkyl.

AutoDockTools-1.5.6 was used for molecular docking simulation to predict the binding mode of Saq B_1_ at the active site of PI3K, which was helpful for molecular biology experiments. Since PI3K γ/δ exists only in immune cells, and the PIK3CA gene encodes the p110α catalytic subunit to form PI3Kα protein, we selected PI3Kα for our study [23]. The optimal binding mode of compound Saq B_1_ with PI3Kα is shown in Figure 4A. The results of docking analysis showed that the lowest binding energy of Saq B_1_ was −8.34 kcal/mol. In addition, we found that Saq B_1_ was located in a favorable position in the PI3Kα domain and binds through a hydrogen-bond and alkyl interaction (Figure 4B). Therefore, it appears that Saq B_1_ inhibits the PI3K/AKT pathway through targeted binding with PI3Kα.

### 2.3. Saq B_1_ Induces Apoptosis in Human CRC Cells by Regulating the PI3K/AKT Pathway

**Figure 5:**
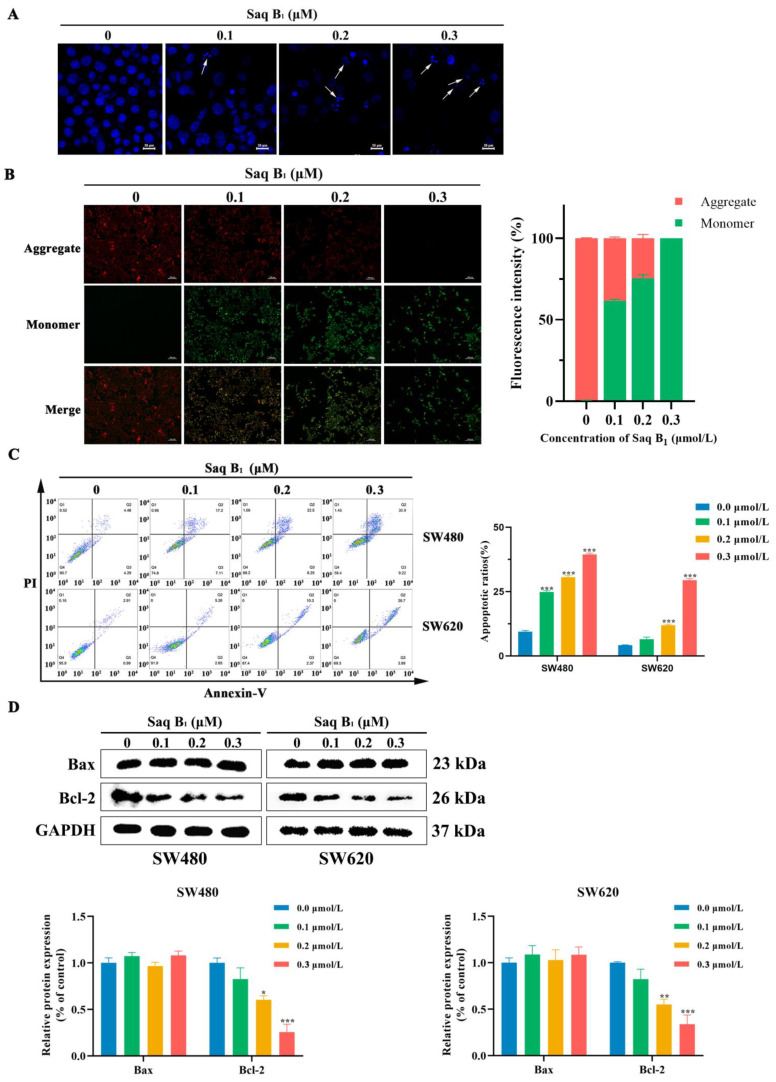
Saq B_1_ induces apoptosis in CRC cells. (**A**) DAPI staining was used to study the effect of Saq B_1_ on apoptosis in SW480 cells. The arrows show nuclei that are condensed and fragmented within the cell. Scale bars = 20 µm. (**B**) Changes in MMP were detected using the JC-1 probe after SW480 cells were treated with different concentrations of Saq B_1_. Representative images are shown. Scale bars = 100 µm. The fluorescence intensity of JC-1 was analyzed using Image J software, and the statistical graph was drawn. (**C)** Apoptosis in SW480 and SW620 cells treated with and without Saq B_1_ was determined by flow cytometry using Annexin V-FITC/PI staining. (**D**) Western blot was used to analyze the expression levels of Bax and Bcl-2. * *p* < 0.05, ** *p* < 0.01, *** *p* < 0.001.

Our previous studies showed that Saq B_1_ might induce apoptosis in CRC cells through the PI3K/AKT signaling pathway. To further investigate whether Saq B_1_ induces CRC cell apoptosis through PI3K/AKT signaling, apoptosis-related experiments were performed. Following DAPI staining of SW480 cells, we found that cells treated with Saq B_1_ showed karyopyknosis, fragmentation of nucleoli, and the formation of apoptotic bodies (Figure 5A). Furthermore, with the increase in Saq B_1_ concentration, the morphologic changes of the cells increased more, but not in untreated cells. JC-1 is an ideal probe for the detection of mitochondrial membrane potential (MMP). When MMP is high, JC-1 exists in the mitochondrial matrix in the form of a polymer, showing red fluorescence. When apoptosis occurs, the MMP is decreased and JC-1 exists in the form of a monomer, producing green fluorescence. The SW480 cells changed from emitting red fluorescence to green fluorescence as the Saq B_1_ concentration increased, indicating that apoptosis had occurred (Figure 5B).

Annexin V-FITC and Propidium Iodide (PI) apoptosis-detection kits were used to determine the specific proportion of apoptotic cells. Annexin V-FITC is used to detect phosphatidylserine, which moves from the inside of the membrane to the surface of the cell membrane during apoptosis, producing green fluorescence. Late apoptotic cells whose cell membrane integrity is destroyed can be stained with PI, producing red fluorescence. Next, after cell staining, we used flow cytometry to analyze Saq B_1_-induced apoptosis and found that as the concentration of Saq B_1_ gradually increased, the content of apoptotic cells gradually increased. The apoptosis rates of SW480 cells were 8.77%, 24.31%, 30.75%, and 40.12%, and the apoptosis rates of SW620 cells were 3.90%, 8.11%, 12.67%, and 30.69%, respectively, for the four concentrations of Saq B_1_ tested (Figure 5C).

The balance between antiapoptotic proteins and proapoptotic proteins in the Bcl-2 protein family is a major determinant of apoptosis. Therefore, the Western blot assay was performed to detect the expression levels of Bcl-2 and Bax proteins in cells changed after treatment with Saq B_1_ at different concentrations. The results showed that the expression of Bax/Bcl-2 increased after treatment in SW480 and SW620 cells (Figure 5D). Thus, we determined that Saq B_1_ initiates apoptosis via the PI3K/AKT signaling pathway in the CRC cell lines SW480 and SW620.

### 2.4. Saq B_1_ Inhibits the Migration and Invasion of Human CRC Cells by Regulating the PI3K/AKT Pathway

**Figure 6:**
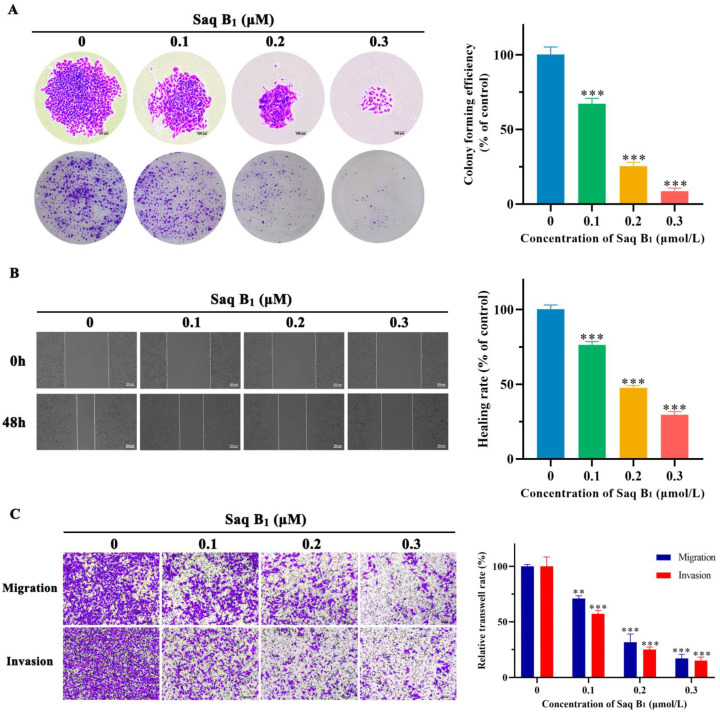
Saq B_1_ suppresses the migratory and invasive ability of SW480 cells. (**A**) The effects of Saq B_1_ on the proliferation of and invasion by SW480 cells were investigated by colony formation assay. The image on the left shows that the size (top) and number (bottom) of colonies formed by cells gradually decreased as the Saq B_1_ concentration increased. Scale bars = 100 µm. The image on the right represents the quantification of the colony-forming efficiency. (**B**) Wound-healing assay to investigate the effects of Saq B_1_ on the migratory abilities of SW480 cells for 48 h. Scale bars = 100 µm. (**C**) Saq B_1_ inhibited the invasive and migratory ability of SW480 cells in the transwell assay. Left: representative images of each condition are displayed. Scale bars = 100 µm. ** *p* < 0.01, *** *p* < 0.001.

The prognosis of patients with CRC is closely related to invasion and metastasis. Once metastasis occurs, treatment will enter the bottleneck stage. Therefore, we verified the ability of Saq B_1_ to inhibit CRC cells invasion and metastasis through a colony formation assay, wound-healing analysis experiments, and a transwell assay. The results of the colony formation assay showed that as Saq B_1_ concentration increased, the proliferative and invasive ability of SW480 cells gradually decreased, and the size and number of cell clones declined (Figure 6A). Treatment with Saq B_1_ inhibited the migration of SW480 as shown by the wound-healing migration assay, indicating that the wound-healing rate of cells decreased gradually as the Saq B_1_ dose increased (Figure 6B). In the transwell trial, Saq B_1_ treatment led to dose-dependent inhibition of the cellular migration and invasion of SW480 cells (Figure 6C). These results collectively suggest the crucial role of Saq B_1_ in retarding the migratory and invasive ability of human CRC cells.

**Figure 7:**
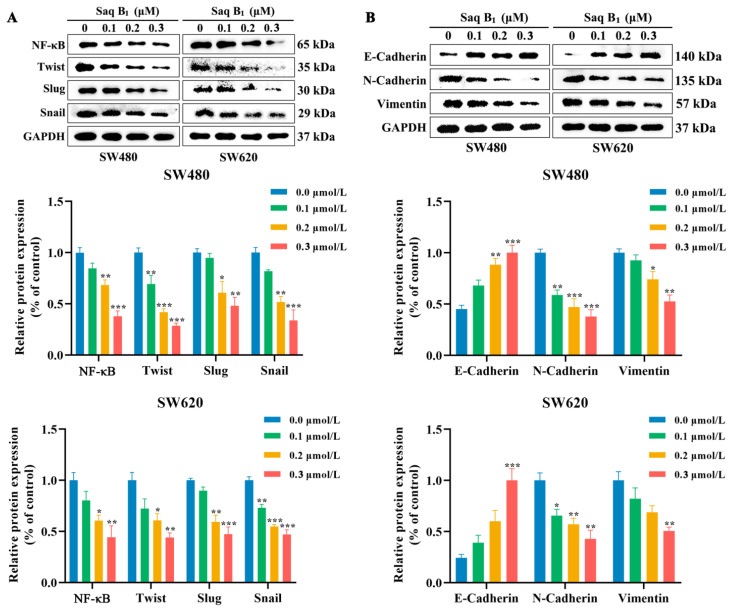

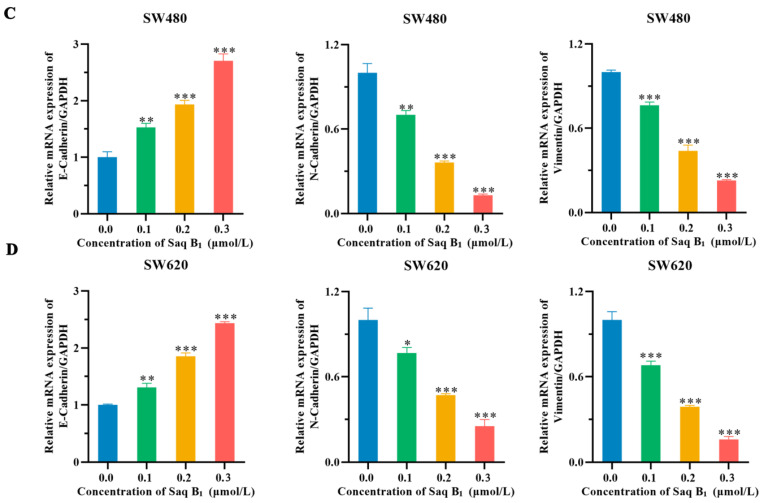
Saq B_1_ affects the expression of EMT-related proteins and mRNA. (**A**) SW480 and SW620 cells were treated with 0–0.3 μmol/L Saq B_1_, then the protein expression of NF-κB, Twist, Slug, and Snail was measured by Western blot. (**B**) Western blot analysis was used to analyze the levels of E-cadherin, N-cadherin, and vimentin. (**C**,**D**) RT-PCR results showed that N-cadherin and vimentin mRNA expression was downregulated and that the mRNA expression of E-cadherin was upregulated after Saq B_1_ treatment compared with the GAPDH expression level. * *p* < 0.05, ** *p* < 0.01, *** *p* < 0.001.

Previous studies have shown that the PI3K/AKT signaling pathway plays an important role in inhibiting invasion and metastasis in CRC cells. In order to further study the mechanism of action of Saq B_1_, Western blotting was used to evaluate the changes in the expression levels of each marker protein in the PI3K/AKT signaling pathway. The protein expression levels of PI3K and p-AKT decreased with the increase in Saq B_1_ concentration. Decreased expression of p-AKT protein resulted in decreased expression of NF-κB and Twist protein, and further led to a decline in the proteins of the snail family transcription repressor 1 (Snail) and snail family transcription repressor 2 (Slug), and ultimately inhibited EMT (Figure 7A). We found that the expression of mesenchymal protein N-cadherin and vimentin decreased as Saq B_1_ concentration increased. In contrast, the expression of epithelial adhesion protein E-cadherin increased (Figure 7B). We also obtained a similar result in the RT-PCR experiment, which demonstrated that E-cadherin gene levels were upregulated, whereas the levels of N-cadherin and vimentin genes were downregulated, which further verified our experimental results (Figure 7C,D). In conclusion, combined with the above data, Saq B_1_ is most likely to induce apoptosis and prevent EMT through the PI3K/AKT signaling pathway in CRC cells.

## 3. Discussion

The occurrence and development of CRC are the result of the joint action of many factors that can result in invasion and metastasis. At present, CRC is mainly treated by surgery and chemical drugs, but the currently available anticancer drugs have strong side effects, and high-efficiency and low-toxicity drugs are urgently needed [24]. Because marine organisms live in a high-pressure, high-salt, hypoxia, and low- (no-) light environment, they contain special substances or metabolites. These molecules have unique and diverse chemical structures and good biological activities, bringing novel inspiration to the development and design of anticancer drugs [25]. In this study, we provide a series of experimental data showing that marine-derived Saq B_1_ can effectively inhibit invasion and metastasis and induce apoptosis in the CRC cell lines SW480 and SW620, with high expression of the *PIK3CA* gene through the PI3K/AKT signaling pathway.

We have previously isolated four angucyclines from the actinomycete *Streptomyces* sp. of marine origin, and their activities are quite different. Comparison of the structure–activity relationship of these angucyclines could provide a basis for purposefully modifying their chemical structure and new ideas for developing angucyclines with better biological activity. Angucyclines have an angular tetracyclic core with different substituents, and in most cases, sugar or amino acid residues are attached to the aglycone skeleton [13]. Through data analysis, we found that the activity of Lan N was far lower than that of the other compounds, which may be due to the conjugation system of the molecule and the influence of polarity. In contrast to Lan N, the angular tetracyclic core of the other compounds has a nonplanar structure with enhanced activity by binding to multiple protein receptors. At the same time, substituents such as hydroxyl groups, carbonyl groups, methyl groups, and sugar groups on the A-ring may influence the polarity and dipole moment. Compared with Mor B, Saq B_1_ and Saq B showed better activity against CRC, which may be due to the introduction of two hydroxyl groups in C-4a and C-12b that can easily form hydrogen bonds with protein receptors or bind to basic groups in protein receptors. Therefore, the introduction of a polar substituent on the A-ring could increase the polarity of the molecule, potentially improving the activity of the compound. However, the introduction of a glycosyl side chain in the C-3 position of Saq B increased steric hindrance and reduced its activity compared with Saq B_1_, as verified by Abdelfattah et al., while the activity of moromycin A (Mor A), which has more steric hindrance, was lower than that of Mor B [26]. Saq B_1_ showed the best activity against CRC among the four angucyclines. Subsequently, we investigated the possible mechanism of action of Saq B_1_ against CRC.

Our current results indicate that the small molecule compound Saq B_1_ has a potential therapeutic window with an IC_50_ of 0.18–0.84 µM for CRC cells and 1.57 µM for noncancerous QSG-7701 cells. PI3Ks can be divided into three categories, with different structures and functions, among which the most widely studied is class I PI3K, which plays an important role in the signal transduction pathway [23]. Among class I PI3K, PI3Kα and PI3Kβ are widely distributed in humans, while PI3Kγ and PI3Kδ are usually highly enriched in leukocyte subtypes [23]. The PI3Kα protein is encoded by the *PIK3CA* gene, so PI3Kα is of great significance as a target in solid tumors. Overactivation of the PI3K/AKT signaling pathway has been reported to play an important role in initiation and progression events such as metastasis, drug resistance, and carcinogenesis of CRC, and is therefore considered a significant therapeutic target [27]. The results showed that Saq B_1_ significantly reduced the expression of PI3K and p-AKT proteins, which was also confirmed by immunofluorescence staining. In addition, the interaction between Saq B_1_ and PI3Kα was detected by molecular docking analysis, which strongly supported the hypothesis that Saq B_1_ might have a targeted inhibitory effect on PI3Kα. Previous studies have reported that urdamycin E, an angucycline from *Streptomyces* sp. OA293, inactivates mTORC1/C2 and induces programmed cancer cell death [28]. At the same time, mTORC1 and mTORC2 complex participate in PI3K/AKT signaling pathway, in which, when mTORC2 protein is activated, it phosphorylates AKT at Ser 473 to inhibit cell apoptosis [29,30]. Our results showed that the expression of p-AKT (Ser 473) protein in SW480 and SW620 cells was decreased after Saq B_1_ treatment, but whether the mTOR pathway was involved needs to be further verified.

EMT is an important mechanism for tumor development and wound healing, but in cancer, it also mediates the progression and spread of aggressive tumors and increases therapeutic resistance [31]. Therefore, effective inhibition of EMT may be one of the key links for the treatment of CRC. It is well-known that the PI3K/AKT signaling pathway promotes cellular EMT processes through the activation of Twist, Snail, and Slug transcription factors [32,33]. The results showed that SW480 and SW620 cells treated with Saq B_1_ significantly reduced the invasion and metastasis of CRC cells, and transcription factors such as Twist, Snail, and Slug were decreased, which further inhibited the expression of downstream EMT-related proteins. EMT is ultimately performed by two independent dynamic events. First, epithelial adhesive protein E-cadherin and junctional proteins are downregulated, resulting in unstable cell adhesion. Second, mesenchymal proteins such as N-cadherin and vimentin are upregulated to drive cell movement and invasion [34]. The results showed that Saq B_1_ successfully increased E-cadherin protein and mRNA, and downregulated N-cadherin and vimentin proteins and mRNA, effectively inhibiting the EMT process.

There are two important apoptotic signaling pathways: the death receptor-mediated pathway (extrinsic) and the mitochondrial-dependent pathway (intrinsic). The PI3K/AKT signaling pathway is closely related to mitochondrial-dependent apoptosis [35]. Studies have found that the *PIK3CA*-based mutation of CRC cell lines can cause resistance to the apoptosis induced by the lack of growth factor due to the activation of the PI3K/AKT signaling pathway. It is highly sensitive to the PI3K inhibitor LY294002, and *PIK3CA*-specific siRNAs can significantly induce apoptosis [36]. According to the results, when Saq B_1_ acts on CRC cells, blocking PI3K activity can reduce the expression of p-AKT in cells [37]. At this point, the increased Bax/Bcl-2 then causes the decrease in MMP, resulting in the release of cytochrome c from mitochondria to the cytoplasm, which then further activates caspases, which can cause chromatin shrinkage, DNA fragmentation, cell lysis, and apoptosis [38,39].

## 4. Materials and Methods

### 4.1. Chemicals and Reagents

Mor B, Saq B_1_, Saq B, and Lan N (purity > 98%) were obtained from Dr. Xie (Shandong University, Weihai, China), who isolated them from marine-derived *Streptomyces* sp. OC1610.4. Doxorubicin was acquired from Sigma-Aldrich Crop. (St. Louis, MO, USA). The compounds were dissolved in dimethyl sulfoxide (DMSO). Antibodies against PI3K, AKT, phosphor-AKT (Ser473), NF-κB, Twist, and GAPDH were purchased from Cell Signaling Technology (CST, Inc., Beverly, MA, USA). Antibodies against Bax, Bcl-2, E-cadherin, N-cadherin, Vimentin, Slug, and Snail were purchased from WanLei Biotechnology (Shenyang, China). The Annexin V-FITC apoptotic detection kit, 5,5′,6,6′-Tetrachloro-1,1′,3,3′-tetraethyl-imidacarbocyanine (JC-1) staining kit, and 4′,6-diamidino-2-phenylindol (DAPI) staining solution were purchased from Beyotime Institute of Biotechnology (Shanghai, China).

### 4.2. Cell Lines and Cell Culture

Human CRC cell lines SW480 (BeNa Culture Collection, Xinyang, China), SW620 (Procell Life Science & Technology Co., Ltd., Wuhan, China), and HT-29 (Zhong Qiao Xin Zhou Biotechnology Co., Ltd., Shanghai, China) were cultured in RPMI 1640 Medium (Livning Biotechnology Co., Ltd., Beijing, China), LoVo cells (Zhong Qiao Xin Zhou Biotechnology Co., Ltd., Shanghai, China) were cultured in DMEM/F12 (Livning Biotechnology Co., Ltd., Beijing, China), and normal human liver cell line QSG-7701 (Beyotime Institute of Biotechnology, Shanghai, China) was cultured in DMEM/high glucose (Livning Biotechnology Co., Ltd., Beijing, China) containing 10% fetal bovine serum (FBS) (Gibco Life Technologies, San Jose, CA, USA). The cells were cultured in a constant temperature incubator at 37 °C, 5% CO_2_.

### 4.3. Cell Cytotoxicity Assay

The cytotoxicity of Mor B, Saq B_1_, Saq B, and Lan N on CRC and the normal human liver cells was detected by MTT (3-(4,5-dimethylthiazol)-2,5-diphenyltetrazolium bromide) assay (Sigma-Aldrich Corp., St. Louis, MO, USA). Cells were evenly distributed in 96-well plates overnight and then treated with Mor B, Saq B_1_, Saq B, and Lan N at different concentrations for 48 h. The negative control group was treated with the same concentration of DMSO. After treatment, 10 μL of the 5 mg/mL MTT solution was added to each well in the incubator for 4 h. Then, the MTT solution was removed and 150 μL DMSO was added into each hole to dissolve the purple crystal. The absorbance of each well was read at 570 nm with a microplate reader, and the IC_50_ values were calculated using the software. Each sample was measured three times independently.

### 4.4. Survival Analysis

The online database Gene Expression Profiling Interactive Analysis (GEPIA) (http://gepia.cancer-pku.cn/, accessed on 24 March 2022) was used to analyze the survival rates of patients with different levels of *PIK3CA* gene expression and draw Kaplan–Meier curves. The data contained 270 cases of patients, which, respectively, expressed a high or low level of *PIK3CA*. The median gene expression was used as the grouping criterion. The samples with higher gene expression than the median were divided into the high-expression group, and the samples with lower gene expression were divided into the low-expression group. Survival analysis was performed on two groups of samples. Parameters related to overall survival are calculated automatically by the website.

### 4.5. Western Blot Analysis 

Western blotting was used to reveal changes in the expression of specific proteins after Saq B_1_ treatment. The cells treated with varying doses of Saq B_1_ were lysed with RIPA buffer (Beyotime Biotech, Shanghai, China) to obtain total protein, which was quantified using a BCA kit. The proteins with different molecular weights were separated by SDS-PAGE electrophoresis. The protein was transferred to a PVDF membrane and sealed with 5% skim milk for 1 h. After washing with TBS-T buffer, the respective primary antibodies were added to the membrane and incubated at 4 °C overnight. Next, the PVDF membrane was washed with TBS-T buffer three times and incubated with antimouse IgG or antirabbit IgG secondary antibody (CST, Inc., Beverly, MA, USA) for 1 h at room temperature. Proteins on the membranes were visualized using the enhanced chemiluminescence detection kit (ECL^®^, Amersham Biosciences, Little Chalfont, UK).

### 4.6. Immunofluorescence Staining

Cellular immunofluorescence was used to observe the localization of the protein in the cell, with the intensity of the fluorescence reflecting the difference in protein expression. The cell suspensions were transferred to a 24-well plate containing 12 mm glass slides and incubated at 37 °C until the cells adhered to the slides, and different concentrations of Saq B_1_ were added. Then, the cells on the glass slides were fixed with 4% paraformaldehyde for 15 min, washed with cold PBS three times, and permeated with 0.1% Triton X-100 at room temperature for 20 min. To prevent nonspecific antibody binding, the cells were washed and incubated with 5% BSA for 1 h. Next, the preparation was incubated with p-AKT antibody overnight at 4 °C and then with FITC goat antirabbit secondary antibody for 1 h. After washing three times with PBS, the samples were dyed with 4 μg/mL DAPI at room temperature for 10 min. Finally, a fluorescence microscope was used to detect and take fluorescence images.

### 4.7. Docking Studies

Molecular docking was used to study the interaction between drug molecules and target proteins and thus to predict their binding patterns and affinity. The PI3K 3D structure was downloaded from Protein Data Bank (PDB code: 3ZIM). AutoDockTools-1.5.6 provided several predictive binding modes through the automatic docking of ligands to targets. The conformation with the lowest binding energy was designated as the most suitable chemical and ligand model. The Lamarckian genetic algorithm was used for docking analysis.

### 4.8. DAPI Staining

Changes in the nucleus were shown by DAPI staining. The cells were seeded into a 24-well plate containing a circular glass sheet and cultured in an incubator until adherent. Different concentrations of Saq B_1_ were added to each well for 24 h; then, the medium was removed and washed with precooled PBS. Next, 4% paraformaldehyde was added for 15 min. The cells were then stained with DAPI (4 μg/mL) for 15 min at 4 °C. The coverslips containing cells were mounted on microscope slides using the antifade mounting medium and analyzed by fluorescence microscopy.

### 4.9. Mitochondrial Membrane Potential Assay

A decline in MMP is a marker event in the early stage of apoptosis. We used the change in color of JC-1 from red to green fluorescence as an early indicator of apoptosis. Specific steps were performed according to the manufacturer’s instructions. In short, the cells were seeded in a 6-well plate, and the medium containing JC-1 was cultured in an incubator for 20 min after Saq B_1_ treatment. The cells were then washed twice with PBS, added to a new medium, and observed under an inverted fluorescence microscope.

### 4.10. Flow Cytometry Analysis of Apoptosis

An Annexin V-FITC apoptosis detection kit was used to detect cell apoptosis by flow cytometry after staining. The cells were placed in 6-well plates and digested by trypsin (Solarbio, Beijing, China) after Saq B_1_ treatment. Then, the cells were washed with PBS and centrifuged for 5 min, and the supernatant was discarded. Annexin V-FITC binding buffer was added to gently suspend the cells. Annexin V-FITC and PI were successively added and slowly mixed. Apoptosis was immediately assessed by flow cytometry (Becton, Dickinson and Company, San Jose, CA, USA). Each sample was measured three times.

### 4.11. Colony Formation Assay

The cell colony formation assay is an important technique for detecting cell proliferation, invasiveness, and drug sensitivity. After digestion of trypsin in the logarithmic growth phase, the cells were inoculated on 6-well plates at a density of 700 cells/well. The medium was refreshed every 3 days and treatment continued for 14 days. Finally, the colonies were fixed with 4% paraformaldehyde and stained with crystal violet. Colonies with >50 cells were counted and photographed.

### 4.12. Wound-Healing Assay

To determine the migratory ability of cells in vitro, we adopted the cell scratch test. Cells were placed in a 6-well plate at a density of 5 × 10^4^ cells per well. When the cells had adhered to 90% of each well, we made equal-width scratches at the same position in each hole with sterile micropipette tips. Each well was washed three times with PBS to remove suspended cells. The cells in the well were treated with different concentrations of Saq B_1_. The wound-healing rate (%) = (1 − (the width of the assay group/the width of the control group)) × 100% was calculated according to the width of the scratches in each group to evaluate the migratory ability of the cells. Each sample was measured three times.

### 4.13. Transwell Assay

The transwell assay is used to test the ability of cells to invade and metastasize. In the invasion assay, we placed transwell chambers in a 24-well plate, and cells were suspended in 200 μL serum-free medium at the density of 1 × 10^5^ cells/mL. Then, cells were added to an upper chamber containing Matrigel, while 700 μL of the medium containing 20% FBS as a chemo-attractant was added to the lower chamber. The cells were treated with various doses of Saq B_1_ and incubated with 5% CO_2_ at 37 °C. Thereafter, all media were discarded, and the cells in the lower chamber were fixed in 4% paraformaldehyde for 20 min and placed in 0.5% crystal violet dye for 20 min. After washing the cells with PBS three times, five regions were selected randomly under the microscope, pictures were taken, and the number of transmembrane cells was counted. The migration assay followed the same procedure as the invasion assay but without Matrigel. Each sample was evaluated independently three times.

### 4.14. RNA Extraction and Quantitative Real-Time PCR

Real-time fluorescence quantitative PCR was used to determine changes in gene expression after Saq B_1_ treatment. Total RNA was extracted from the cells using the RNA easy kit (Sparkjade Biotec Co., Ltd., Shandong, China) according to the manufacturer’s instructions. Then, we converted the RNA into cDNA using the SPARK script II RTPlus kit (Sparkjade Biotec Co., Ltd., Shandong, China). According to the SPARK script II SYBR Green qRT-PCR Kit (Sparkjade Biotec Co., Ltd., Shandong, China) instructions, the template DNA, forward and reverse primers, ddH_2_O, and fluorescent probe were successively added to measure the expression level of related genes. The 2^−∆∆CT^ value was used to analyze the RT-qPCR data. Each sample was evaluated three times independently. The primer sequences used are displayed in Table 2.

### 4.15. Statistical Analysis

The values are presented as the mean ± SEM. Statistical significance for comparisons of three or more groups was determined by one-way analysis of variance (ANOVA). Differences were considered to be statistically significant when the *p*-value was less than 0.05. Significant differences are presented as: * *p* < 0.05; ** *p* < 0.01; *** *p* < 0.001.

## 5. Conclusions

In this study, we analyzed the structure–activity relationship of four angucyclines, providing ideas for the further study of angucyclines. The results show that the angular tetracyclic core of angucyclines is a nonplanar structure, and the introduction of more polar groups in the A-ring may enhance the activity of the compound. It is worth noting that the introduction of larger groups on the A-ring may cause steric hindrance, which reduces the activity of the compound. The results exhibited that Saq B_1_ showed great potential cytotoxic activity on CRC cells compared to other tested compounds. We found for the first time that Saq B_1_ inhibited the proliferative, invasive, and metastatic ability of SW480 and SW620 cells in vitro by regulating the PI3K/AKT signaling pathway and promoting their apoptosis. Although Saq B_1_ has potential anticancer activity, its development as a treatment for CRC by inhibiting the PI3K/AKT pathway needs to be approached with caution. In vivo toxicity tests and later clinical applications may require further research.

## Figures and Tables

**Table 1:** IC_50_ of four angucyclines and doxorubicin in different cell lines at 48 h.

Compounds	SW480	SW620	LoVo	HT-29	QSG-7701
IC_50_ (μmol/L)					
Moromycin B	0.45 ± 0.06	0.34 ± 0.05	0.78 ± 0.02	0.82 ± 0.04	1.26 ± 0.15
Saquayamycin B_1_	0.18 ± 0.01	0.26 ± 0.03	0.63 ± 0.07	0.84 ± 0.05	1.57 ± 0.12
Saquayamycin B	0.32 ± 0.03	0.42 ± 0.02	0.75 ± 0.03	0.89 ± 0.02	1.38 ± 0.14
Landomycin N	>20	>20	>20	>20	>20
Doxorubicin	0.91 ± 0.08	1.06 ± 0.11	0.83 ± 0.12	1.02 ± 0.10	1.33 ± 0.03

Cytotoxicity of four angucyclines and doxorubicin on SW480, SW620, LoVo, HT-29, and QSG-7701 cells were examined by MTT assay. Cells were exposed to varying concentrations of compounds for 48 h and the IC_50_ values were then calculated. Equal volumes of dimethyl sulfoxide (DMSO) were used as the negative control. Data were expressed as the mean ± SD (standard deviation) of triplicate independent experiments.

**Table 2:** The primers used in this study.

Gene	Primer Sequence	
GAPDH	forward primer	5′-CATCAAGAAGGTGGTGAAGCAGG-3′
	reverse primer	5′-TCAAAGGTGGAGGAGTGGTGTCGC-3′
E-cadherin	forward primer	5′-CGAGAGCTACACGTTCACGG-3′
	reverse primer	5′-GGGTGTCGAGGGAAAAATAGG-3′
N-cadherin	forward primer	5′-CCTTTCAAACACAGCCACGG-3′
	reverse primer	5′-TGTTTGGGTCGGTCTGGATG-3′
Vimentin	forward primer	5′-GACGCCATCAACACCGAGTT-3′
	reverse primer	5′-CTTTGTCGTTGGTTAGCTGGT-3′

## Data Availability

The data presented in this study are available on request from the corresponding author.

## References

[1] Sung H., Ferlay J., Siegel R.L., Laversanne M., Soerjomataram I., Jemal A., Bray F. (2021). Global cancer statistics 2020: GLOBOCAN estimates of incidence and mortality worldwide for 36 cancers in 185 countries. CA Cancer J. Clin..

[2] Siegel R.L., Miller K.D., Fuchs H.E., Jemal A. (2022). Cancer statistics, 2022. CA Cancer J. Clin..

[3] Siegel R.L., Miller K.D., Sauer A.G., Fedewa S.A., Butterly L.F., Anderson J.C., Cercek A., Smith R.A., Jemal A. (2020). Colorectal cancer statistics, 2020. CA Cancer J. Clin..

[4] Vatandoust S., Price T.J., Karapetis C.S. (2015). Colorectal cancer: Metastases to a single organ. World J. Gastroenterol..

[5] Benson A.B., Venook A.P., Al-Hawary M.M., Arain M.A., Chen Y.-J., Ciombor K.K., Cohen S., Cooper H.S., Deming D., Garrido-Laguna I. (2020). Rectal Cancer, Version 6.2020 Featured Updates to the NCCN Guidelines. J. Natl. Compr. Cancer Netw..

[6] Ersahin T., Tuncbag N., Cetin-Atalay R. (2015). The PI3K/AKT/mTOR interactive pathway. Mol. Biosyst..

[7] Alzahrani A.S. (2019). PI3K/Akt/mTOR inhibitors in cancer: At the bench and bedside. Semin. Cancer Biol..

[8] Ligresti G., Militello L., Steelman L.S., Cavallaro A., Basile F., Nicoletti F., Stivala F., McCubrey J.A., Libra M. (2009). PIK3CA mutations in human solid tumors Role in sensitivity to various therapeutic approaches. Cell Cycle.

[9] Marmol I., Sanchez-de-Diego C., Pradilla Dieste A., Cerrada E., Jesus Rodriguez Yoldi M. (2017). Colorectal Carcinoma: A General Overview and Future Perspectives in Colorectal Cancer. Int. J. Mol. Sci..

[10] Hoxhaj G., Manning B.D. (2020). The PI3K-AKT network at the interface of oncogenic signalling and cancer metabolism. Nat. Rev. Cancer.

[11] Hua H., Zhang H., Chen J., Wang J., Liu J., Jiang Y. (2021). Targeting Akt in cancer for precision therapy. J. Hematol. Oncol..

[12] Scartozzi M., Giampieri R., Maccaroni E., Mandolesi A., Biagetti S., Alfonsi S., Giustini L., Loretelli C., Faloppi L., Bittoni A. (2012). Phosphorylated AKT and MAPK expression in primary tumours and in corresponding metastases and clinical outcome in colorectal cancer patients receiving irinotecan-cetuximab. J. Transl. Med..

[13] Rohr J., Thiericke R. (1992). Angucycline Group Antibiotics. Nat. Prod. Rep..

[14] Kharel M.K., Pahari P., Shepherd M.D., Tibrewal N., Nybo S.E., Shaaban K.A., Rohr J. (2012). Angucyclines: Biosynthesis, mode-of-action, new natural products, and synthesis. Nat. Prod. Rep..

[15] Qu X.-Y., Ren J.-W., Peng A.-H., Lin S.-Q., Lu D.-D., Du Q.-Q., Liu L., Li X., Li E.-W., Xie W.-D. (2019). Cytotoxic, Anti-Migration, and Anti-Invasion Activities on Breast Cancer Cells of Angucycline Glycosides Isolated from a Marine-Derived *Streptomyces* sp.. Mar. Drugs.

[16] Panchuk R.R., Lehka L.V., Terenzi A., Matselyukh B.P., Rohr J., Jha A.K., Downey T., Kril I.J., Herbacek I., van Schoonhoven S. (2017). Rapid generation of hydrogen peroxide contributes to the complex cell death induction by the angucycline antibiotic landomycin E. Free Radic. Biol. Med..

[17] Hall S.R., Blundon H.L., Ladda M.A., Robertson A.W., Martinez-Farina C.F., Jakeman D.L., Goralski K.B. (2015). Jadomycin breast cancer cytotoxicity is mediated by a copper-dependent, reactive oxygen species-inducing mechanism. Pharmacol. Res. Perspect..

[18] Hall S.R., Toulany J., Bennett L.G., Martinez-Farina C.F., Robertson A.W., Jakeman D.L., Goralski K.B. (2017). Jadomycins Inhibit Type II Topoisomerases and Promote DNA Damage and Apoptosis in Multidrug-Resistant Triple-Negative Breast Cancer Cells. J. Pharmacol. Exp. Ther..

[19] Shaaban K.A., Ahmed T.A., Leggas M., Rohr J. (2012). Saquayamycins G-K, Cytotoxic Angucyclines from *Streptomyces* sp. Including Two Analogues Bearing the Aminosugar Rednose. J. Nat. Prod..

[20] Bao J., He F., Li Y., Fang L., Wang K., Song J., Zhou J., Li Q., Zhang H. (2018). Cytotoxic antibiotic angucyclines and actinomycins from the *Streptomyces* sp. XZHG99T. J. Antibiot..

[21] Peng A., Qu X., Liu F., Li X., Li E., Xie W. (2018). Angucycline Glycosides from an Intertidal Sediments Strain *Streptomyces* sp. and Their Cytotoxic Activity against Hepatoma Carcinoma Cells. Mar. Drugs.

[22] Danielsen S.A., Eide P.W., Nesbakken A., Guren T., Leithe E., Lothe R.A. (2015). Portrait of the PI3K/AKT pathway in colorectal cancer. Biochim. Biophys. Acta-Rev. Cancer.

[23] Vanhaesebroeck B., Perry M.W.D., Brown J.R., Andre F., Okkenhaug K. (2021). PI3K inhibitors are finally coming of age. Nat. Rev. Drug Discov..

[24] Han N., Li J., Li X. (2022). Natural Marine Products: Anti-Colorectal Cancer In Vitro and In Vivo. Mar. Drugs.

[25] Lu S., Wang J., Sheng R., Fang Y., Guo R. (2020). Novel Bioactive Polyketides Isolated from Marine Actinomycetes: An Update Review from 2013 to 2019. Chem. Biodivers..

[26] Abdelfattah M.S., Kharel M.K., Hitron J.A., Baig I., Rohr J. (2008). Moromycins A and B, isolation and structure elucidation of C-glycosylangucycline-type antibiotics from *Streptomyces* sp. KY002. J. Nat. Prod..

[27] Narayanankutty A. (2019). PI3K/Akt/mTOR Pathway as a Therapeutic Target for Colorectal Cancer: A Review of Preclinical and Clinical Evidence. Curr. Drug Targets.

[28] Dan V.M., Vinodh J.S., Sandesh C.J., Sanawar R., Lekshmi A., Kumar R.A., Kumar T.R.S., Marelli U.K., Dastager S.G., Pillai M.R. (2020). Molecular Networking and Whole-Genome Analysis Aid Discovery of an Angucycline That Inactivates mTORC1/C2 and Induces Programmed Cell Death. Acs Chem. Biol..

[29] Copp J., Manning G., Hunter T. (2009). TORC-Specific Phosphorylation of Mammalian Target of Rapamycin (mTOR): Phospho-Ser(2481) Is a Marker for Intact mTOR Signaling Complex 2. Cancer Res..

[30] Sarbassov D.D., Guertin D.A., Ali S.M., Sabatini D.M. (2005). Phosphorylation and regulation of Akt/PKB by the rictor-mTOR complex. Science.

[31] Oliveira T., Hermann E., Lin D., Chowanadisai W., Hull E., Montgomery M. (2021). HDAC inhibition induces EMT and alterations in cellular iron homeostasis to augment ferroptosis sensitivity in SW13 cells. Redox Biol..

[32] Crespo S., Kind M., Arcaro A. (2016). The role of the PI3K/AKT/mTOR pathway in brain tumor metastasis. J. Cancer Metastasis Treat..

[33] Xue G., Restuccia D.F., Lan Q., Hynx D., Dirnhofer S., Hess D., Rueegg C., Hemmings B.A. (2012). Akt/PKB-Mediated Phosphorylation of Twist1 Promotes Tumor Metastasis via Mediating Cross-Talk between PI3K/Akt and TGF-beta Signaling Axes. Cancer Discov..

[34] Zhang N., Ng A.S., Cai S., Li Q., Yang L., Kerr D. (2021). Novel therapeutic strategies: Targeting epithelial-mesenchymal transition in colorectal cancer. Lancet Oncol..

[35] Jiang S., Zhang E., Ruan H., Ma J., Zhao X., Zhu Y., Xie X., Han N., Li J., Zhang H. (2021). Actinomycin V Induces Apoptosis Associated with Mitochondrial and PI3K/AKT Pathways in Human CRC Cells. Mar. Drugs.

[36] Wang J., Kuropatwinski K., Hauser J., Rossi M.R., Zhou Y., Conway A., Kan J.L.C., Gibson N.W., Willson J.K.V., Cowell J.K. (2007). Colon carcinoma cells harboring PIK3CA mutations display resistance to growth factor deprivation induced apoptosis. Mol. Cancer Ther..

[37] Wu Q., Mao Z., Liu J., Huang J., Wang N. (2020). Ligustilide Attenuates Ischemia Reperfusion-Induced Hippocampal Neuronal Apoptosis via Activating the PI3K/Akt Pathway. Front. Pharmacol..

[38] Wang X., Hou Y., Li Q., Li X., Wang W., Ai X., Kuang T., Chen X., Zhang Y., Zhang J. (2019). Rhodiola crenulata attenuates apoptosis and mitochondrial energy metabolism disorder in rats with hypobaric hypoxia-induced brain injury by regulating the HIF-1 alpha/microRNA 210/ISCU1/2(COX10) signaling pathway. J. Ethnopharmacol..

[39] Zhou H., He Y., Zhu J., Lin X., Chen J., Shao C., Wan H., Yang J. (2021). Guhong Injection Protects Against Apoptosis in Cerebral Ischemia by Maintaining Cerebral Microvasculature and Mitochondrial Integrity Through the PI3K/AKT Pathway. Front. Pharmacol..

